# Clonal hematopoiesis of indeterminate potential (CHIP), vascular somatic mutation, and cardiovascular disease: A narrative review

**DOI:** 10.1177/1358863X261437058

**Published:** 2026-08-24

**Authors:** Nicole Powell, Richard M Cubbon, Marc A Bailey, Kathryn J Griffin, Lee Roberts, Chew Cheng

**Affiliations:** 1 Leeds Institute of Cardiovascular and Metabolic Medicine, School of Medicine, Light Laboratories, University of Leeds, Leeds, UK; 2 The Leeds Vascular Institute, Leeds General Infirmary, Leeds Teaching Hospitals NHS Trust, Leeds, UK; 3 Virus Reference Department, Immunisation and Diagnostics Unit, UK Health Security Agency, London, UK

**Keywords:** atherosclerosis, cardiovascular disease, clonal hematopoiesis, endothelial cells, somatic mutations, vascular smooth muscle cells

## Abstract

Somatic cell DNA mutations accumulate with age, and can give cells a survival advantage, resulting in their clonal expansion over time. We know that these mutations can lead to cancer, but the growing application of next-generation sequencing across multiple ‘healthy’ tissues has revealed their ubiquity and relevance to noncancer pathology. Indeed, these mutations may play a role in cardiovascular disease (CVD), particularly in the progression of atherosclerosis and other vascular diseases. This review examines the impact of somatic mutations in both blood and cardiovascular tissues, focusing particularly on clonal hematopoiesis of indeterminate potential (CHIP), and its association with systemic inflammation. CHIP mutations, present in hematopoietic stem cells and their progeny, have been associated with increased risk of CVD, possibly by promoting pro-inflammatory pathways that drive atherosclerosis, although some data suggest CHIP may also arise due to an inflammatory milieu. These somatic variants, and non-CHIP variants, have been detected in atherosclerotic plaques, raising questions about their direct role in atherogenesis and vascular remodeling. Understanding the current research on somatic mutations in blood and cardiovascular tissues may uncover new insights into CVD progression and highlight potential therapeutic targets.

## Introduction

Despite current preventative and therapeutic approaches, cardiovascular disease (CVD) remains the leading global cause of death.^1^

This complex, multifactorial disease is caused by genetic and environmental (e.g., diet, smoking, physical inactivity) factors, and mechanisms such as inflammation and endothelial dysfunction. Inherited (i.e., germline) gene mutations, present in all cells, have a causal role in CVD,^2^ and emerging evidence also implicates later-life nonheritable (i.e., somatic) mutations that develop in individual cells. Some cells with somatic mutations gain a survival advantage and accumulate among ‘normal’ cells (called mosaicism), potentially leading to disease. Cancer is the classical example, but the increasing use of DNA sequencing has found somatic mutation in many diseases, including CVD. This review summarizes literature proposing that somatic mutation in blood and vascular wall cells causes CVD.

## Somatic mutations in blood

A prominent example of mosaicism is clonal hematopoiesis of indeterminate potential (CHIP). This arises from somatic mutations in hematopoietic stem cells (HSCs), which confer a survival advantage, enabling them to outcompete other HSCs. The arising ‘clones’ expand and give rise to mutant circulating immune cells with a pro-inflammatory phenotype. Individuals carrying CHIP mutations experience a > 10-fold greater risk of developing hematological malignancy,^3^ and are at an increased risk of many CVDs (e.g., atherosclerosis, myocardial infarction, and heart failure).^4–7^ In the clinic, CHIP is detected using next-generation sequencing (NGS) of blood DNA, focusing on genes of interest rather than the whole genome. Variants are quantified by variant allele fraction (VAF – the percentage of detected alleles with the specified mutation) as an indicator of clone size. Since blood is readily available and routinely sequenced in people with possible blood disorders, substantial clinical data are available. This led hematologists to discover CHIP as detectable somatic mutations linked to myeloid malignancy, but without evidence of malignancy. Identifying CHIP (or other somatic mutation) in vascular tissues is challenging as biopsy is required, meaning routine clinical data are not available.

## Somatic mutation in cardiovascular tissues

Beyond the blood, somatic mutations have also been detected in vascular tissues and cells, such as vascular smooth muscle cells (VSMCs), and endothelial cells (ECs), which are also important in the pathology of CVDs. Accumulating evidence suggests clonal expansion of somatic mutations (including CHIP variants) in these tissues may contribute to atherosclerosis, through promotion of inflammation, and vascular remodeling and dysfunction.^8–10^ This adds nuance to the notion of atherosclerosis as a disorder of lipid accumulation and systemic inflammation. However, the temporal relationship between somatic mutation and atherogenesis remains incompletely understood.

Somatic mosaicism has also been implicated in nonatherosclerotic vascular disease (e.g., cerebral cavernous malformation [CCM]),^11^ where cerebral lesion formation is driven by somatic mutations in genes such as *PIK3CA*, *KRIT1*, *CCM2*, and *PDCD10*. In addition, recent data have suggested that somatic mutations can arise in the heart.^12^

Despite these emerging insights, many questions remain. This review aims to summarize evidence on the association between somatic mutation, in both the blood and vasculature, and CVD. It will particularly focus on CHIP mutation, given the larger research literature, but non-CHIP somatic mutation will also be considered. Beyond exploring potential causal links between somatic mutation and CVD, the review highlights key knowledge gaps and discusses the potential clinical implications for CVD risk stratification and targeted therapies.

## Clonal hematopoiesis of indeterminate potential (CHIP)

Somatic mutations were first implicated in the development of cancer, but are now known to accrue with age in apparently healthy tissues.^13–15^ Many healthy individuals, particularly with advancing age, harbor myeloid malignancy-associated somatic mutations in their blood or HSCs, but without malignancy or abnormal blood cell counts.^3,16,17^ When present in blood with VAF ⩾ 2%, this is described as CHIP and is associated with increased risk of diverse age-related diseases, including CVD. Immune alterations also characterize ageing, particularly increases in IL-6 and C-reactive protein (CRP), indicating systemic low-grade inflammation.^18^ It is unclear if immune alterations promote ageing or vice versa,^19^ but recent clinical trial data support inflammation directly causing atherosclerotic CVD.^20^ Notably, CHIP does not simply represent age-related genomic instability or cumulative stochastic mutation burden; the growth of clones implies specific mutations provide a survival advantage under particular selection pressures, such as during inflammation.

Recent population studies have uncovered CHIP-driver mutations in many genes, most commonly *DNMT3A*, *TET2*, and *ASXL1*, with less common examples being *JAK2*, *TP53*, *PPM1D*, and *SF3B1L*; many were associated with CVD.^7,21,22^ Some mutations (e.g., nonsense or frameshift variants) cause a nonfunctioning truncated protein, as often seen with *ASXL1* CHIP. Others (e.g., missense variants) alter protein function via single amino acid changes: *DNMT3A* and *TET2* CHIP can rise from missense or nonsense variants. Pich et al. identified over 70 potential CHIP-driver genes, with key roles in epigenetic programming, hematopoietic differentiation, cytokine signaling, and DNA repair.^23^ Epigenetic programming is a process by which expression of many genes is altered by reversible modifications (e.g., methylation) of DNA and/or its histone proteins, with some CHIP genes (e.g., *DNMT3A*, *TET2*) catalyzing these DNA modifications. Epigenetic changes have widespread impacts on cell biology relevant to CVD pathogenesis, such as skewing hematopoietic differentiation toward pro-inflammatory myeloid cells.

## CHIP as a risk factor for cardiovascular disease (CVD)

CHIP has been identified as an independent risk factor for a diverse range of CVDs.^5,7^ Early studies, on small cohorts, reported more than twofold relative risks of CVDs, but subsequent large-scale studies have much smaller (or no evidence of) risk increases, highlighting the need for cautious interpretation of early studies. CHIP is also associated with greater burden of coronary and noncoronary calcification, processes promoted by chronic inflammation that are linked to arterial stiffening and heightened risk of major cardiovascular events.^7,24,25^

Notably, mutations in specific genes may pose different risks of CVD, reflecting the distinct biological roles of these genes and the varying impact of specific mutations on a gene’s expression and activity. For example, *TET2* and *DNMT3A* have been associated with the expansion of atherosclerotic plaques in animal models and humans, possibly through expression of pro-inflammatory cytokines and myeloid cell accumulation.^26–28^ However, although *TET2* mutations confer a > 1.5-fold higher risk of coronary heart disease, *DNMT3A* confers a smaller relative risk. CHIP involving *TP53*, which participates in the DNA-damage response, is associated with a more than twofold greater risk of atherosclerotic CVD events, with mouse models revealing increased plaque macrophage proliferation.^29^

*JAK2* mutant CHIP is linked to a more than twofold higher risk of arterial (e.g., myocardial infarction) and venous thrombotic events, yet paradoxically also increased bleeding events, especially at higher VAF.^30–32^ *JAK2*^V617F^ (a mutation switching valine to phenylalanine at amino acid 617, leading to JAK2 kinase activation) is a key driver in myeloproliferative neoplasms, such as essential thrombocythemia (ET). Murine models suggest this mutation directly induces production of immature hyper-reactive platelets (i.e., increased agonist-induced aggregation and in vivo thrombosis), but also indirectly activates nonmutant platelets via secreted factors.^31^ The bleeding risk may reflect associated acquired von Willebrand factor deficiency, but more research is needed.^32^ Chromosome 9p21.3, a common germline risk locus for CVD, has also been associated with platelet reactivity.^33,34^ Notably, the *CDKN2A* gene resides here, and somatic mutation of this gene is reported in cancer, although there is currently no evidence that somatic mutation of this gene contributes to CHIP or CVD.^35^

Table 1 summarizes reported risks of CVD associated with key CHIP-associated genes. Beyond the differing biology of CHIP genes and mutations, conflicting data may reflect differences in study methodology, such as sequencing depth, population demographics, and VAF thresholds used to define CHIP. These factors should be addressed in future studies to more confidently define CHIP’s role as a CVD biomarker.

**Table 1.** Quantified risks of specific cardiovascular outcomes associated with CHIP mutations.

Mutation	Type of mutation	Mechanism	Cell type	Clinical phenotype
*DNMT3A*	Missense (var R882) + nonsense	Epigenetic dysregulation can lead to altered myeloid differentiation, resulting in increased expression of pro-inflammatory cytokines	Monocytes/macrophages (+ T-cell effects reported)	Increased ASCVD risk: HR 1.70 (1.1–2.6), *p* = 0.01;^7^ cardiac fibrosis; heart failure;^5,6,58^ stroke (HR 1.44, *p* = 0.03);^63^ mortality after TAVR.^54^
*TET2*	Missense + nonsense	Epigenetic dysregulation (reduced methylation), skewing hematopoietic differentiation toward pro-inflammatory myeloid cells	Myeloid cells (monocytes/macrophages)	Increased ASCVD risk: HR 1.90 (1.0–3.7), *p* = 0.06;^7^ HF (HR 1.59, 1.18–2.14);^5,57,61,95^ aortic stenosis; mortality after TAVR;^54^ stroke (HR 1.85, *p* = 0.004);^63^ differential treatment response to antiinflammatory therapies.
*ASXL1*	Nonsense, loss-of-function	Chromatic regulation disruption	Myeloid cells	Increased ASCVD risk: HR 2.0 (1.0–3.9), *p* = 0.05;^7^ HF (HR 1.58, 1.20–2.08);^61,95^ risk is often weaker than TET2/DNMT3A.
*JAK2*	Missense (var V617F)	Altered JAK/STAT signaling, platelet/megakaryocyte reprogramming	Megakaryocytes/platelets/neutrophils	ASCVD: HR 12 (3.8–38), *p* < 0.0001;^7^ thrombosis (*JAK2*^V617F^);^30,31^ MI/stroke; VTE (splanchnic vein thrombosis^96^); bleeding/platelet dysfunction; HF (HR 2.50, 1.35–4.64);^61,95^ *JAK2*^V617F^-specific increased risk of cardiovascular events: HR 3.35 (1.22–9.21), *p* = 0.019.^66^
*TP53*	Missense + nonsense	Macrophages	Impaired DNA damage response	Increased ASCVD/CAD risk: HR 2.31 (1.03–5.16), *p* = 0.042; increased PAD risk: HR 4.98 (1.23–20.09), *p* = 0.024;^29^ mechanisms and associations less established.

Risk estimates reported in studies were defined using HR with a 95% CI or *p*-value.

ASCVD, atherosclerotic cardiovascular disease; CAD, coronary artery disease; CHIP, clonal hematopoiesis of indeterminate potential; HF, heart failure; HR, hazard ratio; JAK/STAT, Janus kinase/signal transducer and activator of transcription; MI, myocardial infarction; PAD, peripheral artery disease; TAVR, transcatheter aortic valve replacement; var, variant; VTE, venous thromboembolism.

## Inflammation as a mechanism connecting CHIP and CVD

The link between CHIP and CVD is often attributed to an inflammatory state. Somatic mutations in HSCs can promote clonal expansion of pro-inflammatory leukocytes, and cytokine production (e.g., IL-1, IL-6), altering vascular homeostasis.^4,36–38^ CHIP promotes inflammation through gene-specific effects.^7,39^ For example, *GNB1* is a direct regulator of the NLRP3 inflammasome, whereas *DNMT3A* and *TET2* have broad-based epigenetic effects.^40^ Larger clone sizes, often seen with *DNMT3A* and *TET2* CHIP, are linked to increased severity of disease.^41,42^ The functional effects of smaller clones (VAF 0.1–2.0) is less clear and requires further research.^42,43^ Notably, CHIP is associated with nonatherosclerotic inflammatory CVDs, such as myopericarditis, and noncardiovascular inflammatory diseases, emphasizing its broad relevance.^44^

It is also reported that inflammation may drive CHIP and growth of CHIP clones, rather than vice versa.^45^ This could imply that CHIP is simply an epiphenomenon in an inflammatory milieu, rather than directly driving CVD. Some studies have used Mendelian-randomization to infer causal associations between CHIP and CVD.^46^ These inferred causal links between CHIP and atrial fibrillation, with weaker evidence for adverse myocardial remodeling, but no link to atherosclerotic CVD events was elicited. Other data show baseline CHIP is associated with future de novo femoral atheroma development, whereas neither baseline atherosclerosis nor atheroma volume is associated with future CHIP clone expansion.^47^ These findings alone cannot confirm or refute causal relationships in isolation and make important assumptions that are difficult to validate. Further mechanistic research is needed to address this issue at the crux of whether CHIP is a therapeutic target for CVD prevention.

## Inflammatory pathways linking CHIP to CVD

### Ten-eleven translocation 2 (*TET2*)

*TET2* is an epigenetic regulator that demethylates DNA, promoting gene expression. *TET2* was one of the first described CHIP-associated genes, and has been repeatedly linked to CVD risk.^39,48–50^ Fuster et al. used competitive bone marrow transplantation in low-density lipoprotein receptor (LDLR)-knockout mice to investigate the role of *TET2*-deficient HSCs in atherosclerosis.^51^ They observed a 60% increase in aortic root plaque size with *TET2* deficiency increasing interleukin-1 beta (IL-1β) expression and NLRP3 inflammasome activation, offering potential mechanistic insight into the atherogenic effect of *TET2* CHIP.^51^ In support, post hoc analysis of clinical trial data has shown *TET2* CHIP is associated with a larger reduction in cardiovascular events from the anti-IL-1β antibody canakinumab.^52^ Beyond atherosclerosis, murine hematopoietic *TET2* loss is associated with accelerated heart failure.^39^ Clinically, *TET2* CHIP is associated with higher mortality after transcatheter aortic valve replacement (TAVR) for severe aortic stenosis, and a longer hospitalization after coronary artery bypass grafting.^53^–^55^

### DNA methyltransferase 3A (DNMT3A)

Conversely, *DNMT3A* is an epigenetic regulator that adds a methyl group to DNA, resulting in gene silencing. *DNMT3A* is the most frequently mutated gene in CHIP and is again associated with atherosclerotic CVD, adverse heart failure outcomes, and mortality after TAVR.^6,7,16,56–58^

Abplanalp et al. showed that *DNMT3A*-mutant monocytes have increased expression of pro-inflammatory cytokines, such as IL-1β and Il-6, and activate T cells in patients with heart failure.^58^ Shumliakivska et al. showed that *DNMT3A*-deficient monocytes activate cardiac fibroblasts, promoting cardiac fibrosis and heart failure progression.^59^

### Other driver genes

Although *TET2* and *DNMT3A* dominate in frequency, many other CHIP genes are associated with CVD risk, including *ASXL1*, *JAK2*, *TP53*, and others.^7,16,60–64^ Kiefer et al. studied CHIP mutations with VAF > 0.5% in patients with ischemic heart failure, and found *CBL*, *CEBPA*, *EZH2*, *GNB1*, *PHF6*, *SMC1A*, and *SRSF2* were significantly associated with increased mortality.^65^ Although mechanistic insights are limited for these genes, pathway enrichment analysis suggested roles in immune regulation and cytokine production.

As noted earlier, *JAK2* mutants contribute to CHIP and myeloproliferative neoplasms, such as polycythemia vera or essential thrombocythemia, with the *JAK2*^V617F^ variant being an independent risk factor for CVD.^66^ *JAK2* normally conveys signals from activated type 2 cytokine receptors; the V617F mutant constitutively activates its kinase, increasing onward signaling to STAT transcription factors, altering gene expression akin to pro-inflammatory cytokine signalling.^67^

### Endothelial cell (EC) somatic mutations and CVD

CHIP-associated low-grade inflammation presumably aggravates atherosclerosis through effects on established plaque, but it is also likely to drive early atherogenesis via endothelial dysfunction. ECs line the vasculature, regulating vascular tone, inflammatory cell adhesion, and thrombosis, among other roles key to health and disease. Emerging evidence suggests somatic mutation may occur in atheroma-associated ECs, with one study finding *JAK2*^V617F^ in both peripheral blood mononuclear cells (PBMCs) and ECs from carotid atherosclerotic plaque.^4,68^ Mouse models of endothelial *JAK2*^V617F^ mutation exhibit systemic disease including cardiomyopathy, along with increased EC and leukocyte-endothelial adhesion, and a pro-thrombotic and inflammatory state.^69,70^ A murine model of myeloid *JAK2*^V617F^ expression also demonstrated loss of endothelial barrier integrity and EC cell apoptosis, illustrating how *JAK2*^V617F^ has both cell autonomous and nonautonomous adverse effects on the endothelium.^71^ *TET2*, a frequently mutated gene in CHIP, has also been implicated in contributing to endothelial dysfunction. *TET2* regulates autophagy, a process which is critical for cellular homeostasis.^72^ Under low shear stress, EC autophagy was found to be reduced through *TET2* downregulation.^73^ Conversely, restoring *TET2* can improve EC function by increasing autophagy.^74^ However, it is yet to be determined whether *TET2* somatic mutation occurs in ECs, and, overall, the data supporting EC somatic mutation in atherosclerosis should be seen as preliminary.

### Vascular smooth muscle cell somatic mutation and CVD

Besides blood and ECs, somatic mutation in vascular smooth muscle cells (VSMCs) is becoming recognized as a potential contributor to atherosclerosis, given their key role in plaque biology. VSMCs can clonally expand, switch phenotypes, and may either stabilize or destabilize plaques.^75^ This nature was first suggested by Benditt and Benditt, who proposed their clonality in atherosclerotic plaques related to somatic mutation.^76^ Subsequent work has supported this theory, with lineage-tracing studies in mice showing that a small subset of VSMCs clonally expand after vascular injury.^77,78^ Likewise, Jacobsen et al. showed that specific VSMC clones contribute to different regions within plaque (e.g., fibrous cap).^79^ Wang et al. used a lineage-tracing model to show that a subset of mature hyper-proliferative VSMCs promote a pro-inflammatory environment through activation of the complement cascade.^8^ These clonal, pro-inflammatory cells gained a survival advantage, which the authors showed was via evasion of phagocytosis by macrophages, which could be reversed by blockade of CD47. Evidently, VSMCs demonstrate great plasticity, and can even differentiate into fibrous-cap-stabilizing ACTA2+ cells or trans-differentiate into chondrocyte-like or macrophage-like cells.^80^ Somatic mutation could promote clonal plasticity, but other mechanisms such as nonmutant vascular wall-resident progenitors and local environmental conditions may play a role, so this requires further investigation.

*TET2* is a regulator of proliferation and phenotypic transformation of VSMCs. VSMC loss of *TET2* in human cell culture and mouse models leads to dedifferentiation, a process in which their contractile state switches to a pro-inflammatory and pro-migratory phenotype, as observed in atherosclerosis. Conversely, *TET2* overexpression promoted their contractile properties.^81^ The same work showed diminished *TET2* expression in human atherosclerotic plaque but did not address if this was clonal.^81^

A recent study reported that CHIP mutations (including in *TET2* and *DNMT3A*) were detectable in blood, atherosclerotic plaque, arterial collaterals, perivascular fat, and subcutaneous tissue from patients with peripheral artery disease undergoing vascular surgery.^82^ The VAFs in these tissues matched or surpassed those in the blood. However, these data may reflect mutant clones from the blood infiltrating the vascular wall rather than somatic mutation in vascular wall-resident cells, such as VSMC.

More recently, whole exome sequencing of carotid plaques revealed somatic mutation in many genes, with locally expanded clonal populations.^83^ Somatic mutations were detected in 12 out of 13 investigated plaques, yet none were detected in 11 nonatherosclerotic arterial tissue samples. VAFs of 1–30% were observed, with some clones spanning as far as 16 mm across both medial and intimal layers. Functional enrichment analyses of mutated genes highlighted contractile function and showed the greatest expression in VSMCs using single-cell transcriptomic data. Six patients had CHIP present in their blood, with the same mutations often found in paired plaques.^83^ Although these findings are interesting, the cellular origins of these mutants and their functional implications are unknown and require further research.

Collectively, these findings highlight the complexity of VSMCs, and the potential for somatic mutations to promote their clonal expansion and pathogenic phenotype transitions. However, further studies are needed to determine whether somatic mutation occurs in VSMCs and if this plays a causal role in CVD. Indeed, it is also conceivable that somatic mutations could be adaptive and protect against adverse outcomes in some circumstances.

### Crosstalk between blood and vascular somatic mutations in CVD

As noted earlier, CHIP leukocytes exhibit altered paracrine signaling and direct cell-cell contacts with a range of cell lineages relevant to CVD, which may represent an important element of their pathogenicity and a therapeutic opportunity. For example, *DNMT3A*-deficient monocytes have been shown to activate cardiac fibroblasts, which promotes cardiac fibrosis and heart failure.^59^ *JAK2*^V617F^ mutant myeloid cells were similarly found to adversely alter the biology of ECs, contributing to thrombosis.^71^ In a murine bone marrow transplant model with 1.5% mutant *JAK2* bone marrow cells and 98.5% wild-type cells, altered signaling between mutant and nonmutant leukocytes was observed.

Increased IL-1β paracrine signaling from mutant myeloid cells was observed, which modulated nonmutant aortic macrophages to promote inflammation, release of neutrophil extracellular traps (NETosis), and plaque growth.^84^ As noted earlier, paracrine activity in *JAK2*^V617F^ also alters the function of nonmutant platelets.^32^ Hence, altered paracrine activity arising from CHIP-mutant leukocytes, even at a modest VAF, can have wide-ranging implications for multiple cell lineages relevant to CVD.

There is currently no evidence that paracrine signaling from leukocytes may cause somatic mutations in vascular cell walls, but this inflammatory milieu could induce a selective advantage that permits clonal growth of mutant vascular wall cells. This could explain similar cross-tissue clonal mutation profiles, although other possibilities, such as leukocyte DNA contamination in vascular wall analyses, need to be considered. This paradigm is summarized in Figure 1, which illustrates how CHIP may directly and indirectly contribute to CVD through its coordinated effects on multiple cell lineages. Figure 1 also illustrates how a somatic mutation in nonleukocyte vascular wall cells could contribute to CVD, although direct evidence for this remains limited and such mutations may not necessarily be in CHIP-associated genes.

**Figure 1.**
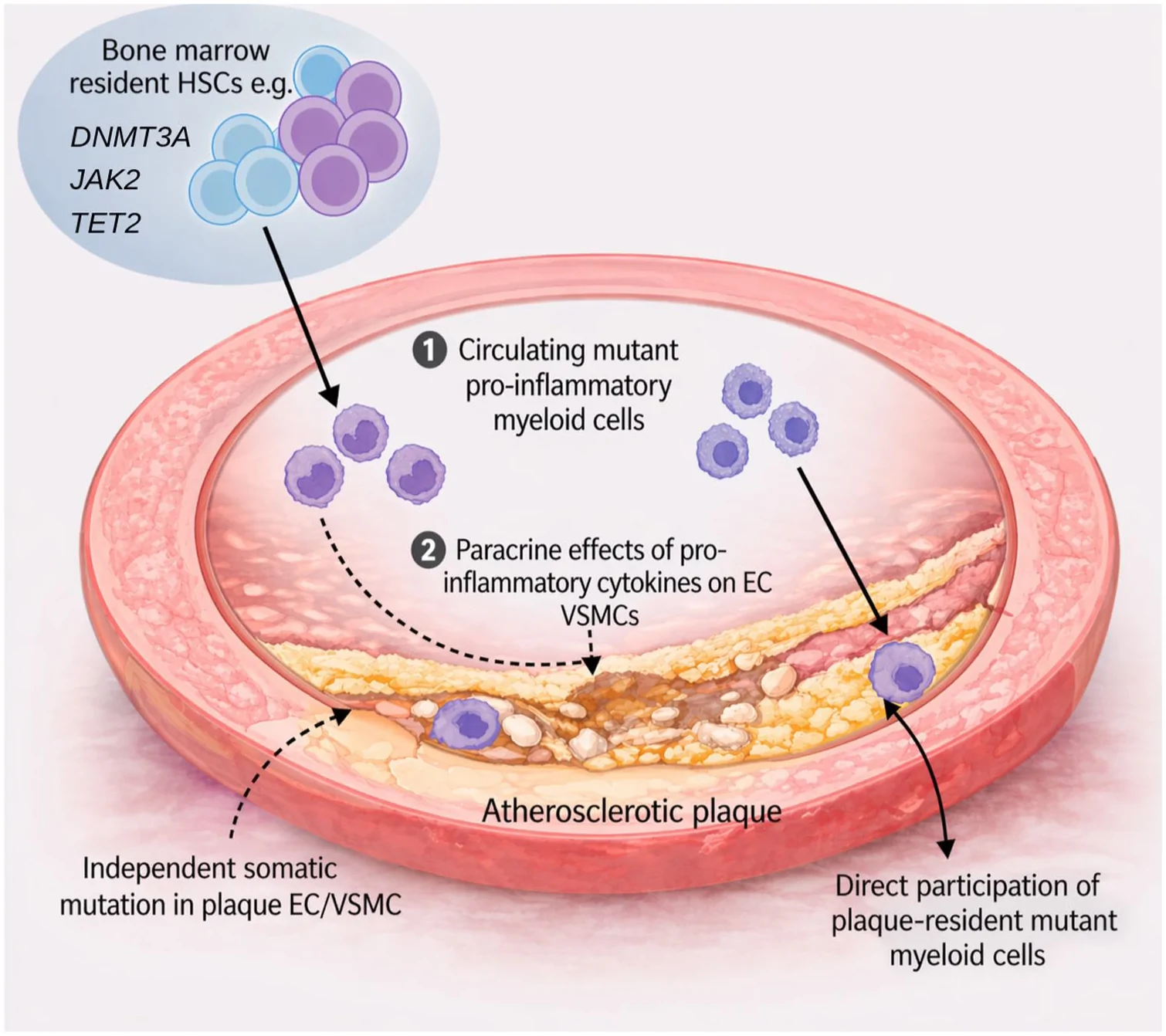
The integrated mechanisms linking somatic mutation in blood and nonblood cells to cardiovascular disease. Somatic mutations may contribute to CVD across multiple cell types. Somatic mutations in HSCs – particularly CHIP-driver genes such as *TET2*, *DNMT3A*, or *JAK2* – can confer a selective advantage and lead to clonal expansion. These mutant clones give rise to pro-inflammatory myeloid cells, which can circulate in the blood and release pro-inflammatory cytokines (e.g., IL-1β, IL-6, and TNFα). There is some evidence that these mutations may be present in ECs and VSMCs, which may contribute to EC dysfunction, or VSMC phenotypic switching. Crosstalk between ECs and immune cells via cytokines has also been suggested. These mechanisms could promote plaque development and progression. CHIP, clonal hematopoiesis of indeterminate potential; CVD, cardiovascular disease; DNMT3A, DNA methyltransferase 3A; EC, endothelial cell; HSC, hematopoietic stem cell; IL-1β, interleukin-1 beta; IL-6, interleukin-6; JAK2, Janus kinase 2; TET2, ten-eleven translocation 2; TNFα, tumor necrosis factor alpha; VSMC, vascular smooth muscle cell. Figure generated using chatGPT-5. Figure is in color online.

Crosstalk between mutant leukocytes, and mutant or nonmutant ECs and VSMCs, may re-enforce inflammation, enhance immune cell recruitment, and facilitate lipid infiltration, driving plaque development. Such a paradigm involving mutant and nonmutant circulating and tissue-resident cells could be relevant to other CVDs; for example, myocardial diseases like heart failure.

## Clinical implications and future directions

Somatic mutation holds significant potential in cardiovascular clinical practice, both in risk stratification and in therapeutic targeting. Building on the mechanistic insights from somatic mutations in both blood and vascular tissues, this section explores their translational potential.

### Biomarkers for risk stratification

There is emerging evidence that somatic mutations could be used as biomarkers for CVD risk. CHIP mutations have already shown potential in predicting cardiovascular events.^41,42,54,57,85–87^ In 13,129 UK Biobank participants with established atherosclerotic cardiovascular disease (ASCVD), 5% were CHIP-carriers defined by DNA exome sequencing;^88^ these had a significantly higher risk of future ASCVD events and all-cause mortality compared to those without CHIP. Larger CHIP clones and multiple large CHIP-driver mutations amplified risk.

Somatic mutations in genes such as *TET2*, *SF3B1*, *SRSF2*, and *U2AF1* were especially predictive of adverse CVD outcomes, and, interestingly, mutations in genes *PPM1D* and *TP53* were strongly associated with mortality in women.^88^

Although most evidence focuses on blood-derived mutations, these same principles of clone size, mutation burden, and gene-specific effects are likely relevant for somatic mutations in vascular tissues. However, the challenges of routinely accessing vascular tissue make blood biomarkers more practical. Notably, emerging technologies profiling circulating cell-free DNA can infer its cellular origin based on methylation status, suggesting 10% of reads arise from the vascular endothelium,^89^ although further research is needed to explore the potential of this and other approaches. Recent advances in artificial intelligence (AI) have also improved the ability to discern clonal hematopoiesis variants from those arising from solid organ tumors using circulating cell-free DNA.^90^ This approach could conceivably extend to the detection of somatic variants arising from CVD. Such findings demonstrate the compelling potential of somatic mutations to be used as biomarkers for early detection and risk stratification. It is also important to emphasize the need for high sequencing depth (i.e., how many examples of each region of DNA is examined) to gain adequate sensitivity and specificity in clinical use.

Table 2 summarizes the current experimental approaches used to detect somatic mutation and clonal hematopoiesis, along with their applications and key limitations.

**Table 2.** Experimental approaches summarized by their primary use (broad discovery vs targeted detection vs validation/quantification), and their ability to detect low variant allele fraction clones.

Technique	Use
WES or WGS	Used for variant discovery but less sensitive at detecting variants with a low VAF due to sequencing depth
Targeted NGS (gene panels/amplicon sequencing)	Target next-generation sequencing of CHIP panels with greater depth to detect variants with low VAFs
Droplet digital PCR (ddPCR)	Nonsequencing-based technique to quantify VAF of a known variant; not used for discovery of new variants

CHIP, hematopoiesis of indeterminate potential; NGS, next-generation sequencing; PCR, polymerase chain reaction; VAF, variant allele fraction; WES, whole-exome sequencing; WGS, whole-genome sequencing.

### Personalized medicine

Personalized medicine advances risk prediction by using this to define people who are more or less likely to benefit from (or be harmed by) an intervention. Somatic mutation profiling holds potential in this regard, with early data supporting the planning of larger trials in people with CHIP. For example, as discussed earlier, patients with atherosclerosis appear to derive larger benefits from the antiinflammatory therapy canakinumab in the presence of *TET2*-mutant versus non-*TET2*-mutant CHIP. Furthermore, clonal expansion of somatic mutations reflects local and/or systemic selection pressures that are likely to be relevant to the underlying disease process and be amenable to therapeutic modulation. Although the feasibility of this requires further assessment, data from preclinical models, human in vitro and human epidemiological data suggest that metformin can suppress the competitive advantage of *DNMT3A*^R882H^-mutant CHIP.

### Therapeutic targeting

Inflammation is now recognized as a principal mechanism and emerging therapeutic target in ASCVD. The Canakinumab Anti-inflammatory Thrombosis Outcomes Study (CANTOS) showed that inhibiting IL-1β with canakinumab significantly reduced incidence of major cardiovascular events in patients with ASCVD.^20^ As noted above, a post hoc analysis of CANTOS suggested these benefits are greater in patients with *TET2*-mutant CHIP, although this requires validation.

Colchicine, a low-cost, antiinflammatory drug, has shown promise in some,^91,92^ but not all,^93^ ASCVD clinical trials. In atherosclerosis-prone mice with *TET2*-mutant clonal hematopoiesis, colchicine reduced atherosclerosis and IL-1β expression.^28^ In large human cohorts, colchicine use was associated with diminished myocardial infarction rates in people with *TET2*-mutant clonal hematopoiesis.^28^

Post hoc analysis of the LoDoCo2 clinical trial that randomized people with ASCVD to colchicine, found reduced expansion of *TET2* CHIP clones compared with rates in placebo recipients.^94^ These findings suggest that CHIP-driven inflammation represents a therapeutic target in CVD.

Ongoing observational and interventional studies are now underway to assess CHIP-associated cardiovascular risk and to test whether CHIP-informed approaches can guide antiinflammatory therapies. Table 3 summarizes ongoing studies registered with ClinicalTrials.gov in this field.

**Table 3.** Active studies investigating somatic mutation/clonal hematopoiesis and cardiovascular disease according to ClinicalTrials.gov.

Study	ClinicalTrials.gov ID	Study type	Objective
CHIP/CCUS Natural History Protocol	NCT04102423	Observational	Verify the association of myeloid somatic mutations with atherosclerosis and blood cancers and find new potential clinical associations
Ropeginterferon for High Risk *JAK2* Clonal Hematopoiesis	NCT07249840	Phase 1 interventional	Test the feasibility and safety of the drug Ropeginterferon alfa-2b in the treatment of patients with *JAK2* mutation and high-risk features, in the absence of myeloproliferative neoplasm
RICO-HF	NCT06626685	Observational trial; acute and chronic HF cohorts	Investigate the role of CHIP in inflammation, and study the relevance of inflammatory signaling by immune cells in cardiac dysfunction and HF
CHIVE	NCT06701214	Observational	Contribute to the understanding of cardiovascular phenotypes and CHIP, and better characterize clinical outcomes and co-morbidities in CHIP-carriers
CHAPTER	NCT07313059	Observational	Assess the risk of CVD, blood cancers, and personalized care in patients with CHIP
CHIP-RETENTION	NCT04987268	Observational	Identify the association of CHIP and residual cardiovascular risk after smoking cessation
CLODETTE	NCT05711173	Interventional	Ascertain the prevalence of CHIP and/or increased NETosis formation in patients with venous thrombosis
TECTONIC	NCT06691217	Phase 2 interventional	Test the efficacy of the drug canakinumab, an inhibitor of IL-1β, on vascular inflammation in individuals with coronary artery disease
Genetic Test Based Risk Prediction of Early Calcific Aortic Valve Disease in Patients with Bicuspid Aortic Valve	NCT06153407	Observational	Determine the impact of CHIP on the progression of early aortic valve calcification in patients with bicuspid aortic valves
Effect of Colchicine on Progression of Coronary Atherosclerosis in Patients with *TET2*-CHIP Variant	NCT07362966	Phase 4 interventional	Elucidate whether *TET2*-CHIP can be used as a biomarker to guide precision-use of the drug colchicine in patients with coronary atherosclerosis, and if *TET2* status is an indicator of response to colchicine
CHiEF	NCT05828888	Observational	Determine the link between CHIP in endothelial dysfunction and heart failure

Trials are summarized by their registry ID, design (observational vs interventional, and phase where applicable), and primary objective.

CCUS, clonal cytopenia of undetermined significance; CHIP, clonal hematopoiesis of indeterminate potential; CVD, cardiovascular disease; HF, heart failure; IL-1β, interleukin-1 beta; *JAK2*, Janus kinase 2; NETosis, neutrophil extracellular trap; *TET2*, ten-eleven translocation 2.

### Unanswered questions

Though current somatic mutation and clonal hematopoiesis research is promising, it remains in its infancy. The clonal expansion of somatic mutations in the blood is well characterized, but there are sparse data surrounding vascular cell somatic mutations.

Large-scale longitudinal studies that compare the blood and vascular tissue are needed to determine how common vascular somatic mutations are, how they develop over time, and their association with CVD. Another key uncertainty is whether somatic mutations are causally associated with CVD (or vice versa), and if so whether they represent adaptive or maladaptive responses. Although this knowledge is not essential to inform risk prediction, it is paramount for successfully guiding therapeutic approaches.

Finally, although therapeutic hypotheses have focused on CHIP-driven inflammation in the blood, it is unclear whether these therapies would address the pathological effects of vascular somatic mutations. Much more fundamental research is needed to define whether such hypotheses are valid regarding the vascular wall and therapies directed there.

## Summary

CVD is the leading cause of death worldwide, and novel methods of prevention, early detection, and treatment are critical. The recent finding that somatic mutations are prevalent in the blood and vascular wall have opened a promising avenue of translational research.

Somatic mutations accumulate with age and are ubiquitous. Some mutations in HSCs can give rise to CHIP and are an independent risk factor for CVD, possibly via enhanced inflammation. Though originally thought confined to blood, CHIP-associated (and other) mutations are also present in vascular tissue, potentially giving a new perspective on CVD development.

Somatic mutations in vascular cells represent a further layer of disease complexity and an opportunity in translational CVD research. However, many important questions remain about their prevalence and implications. Understanding these might offer important insights into risk stratification, disease mechanisms, and personalized medicine.
